# Prognostic and immunological characteristics of CDK1 in lung adenocarcinoma: A systematic analysis

**DOI:** 10.3389/fonc.2023.1128443

**Published:** 2023-03-06

**Authors:** Qingwu Du, Wenting Liu, Ting Mei, Jingya Wang, Tingting Qin, Dingzhi Huang

**Affiliations:** Department of Thoracic Oncology, Tianjin Medical University Cancer Institute and Hospital, National Clinical Research Center for Cancer, Tianjin Key Laboratory of Cancer Prevention and Therapy, Tianjin’s Clinical Research Center for Cancer, Tianjin, China

**Keywords:** CDK1, lung adenocarcinoma, tumor immune microenvironment, biomarker, prognosis

## Abstract

**Background:**

Cyclin-dependent kinases (CDKs) play a key role in cell proliferation in lung adenocarcinoma (LUAD). Comprehensive analysis of CDKs to elucidate their clinical significance and interactions with the tumor immune microenvironment is needed.

**Methods:**

RNA expression, somatic mutation, copy number variation, and single-cell RNA sequencing data were downloaded from public datasets. First, we comprehensively evaluated the expression profile and prognostic characteristics of 26 CDKs in LUAD, and CDK1 was selected as a candidate for further analysis. Then, a systematic analysis was performed to explore the relationships of CDK1 with clinical characteristics and tumor immune microenvironment factors in LUAD.

**Results:**

CDK1 was markedly upregulated at both the mRNA and protein level in LUAD. Moreover, overexpression of CDK1 was related to poor clinical outcomes. CDK1 coexpressed genes were mainly involved in the cell cycle, the DNA repair process, and the p53 signaling pathway. In addition, CDK1 expression was found to be correlated with the expression of multiple immunomodulators and chemokines, which participate in activating and suppressing the immune microenvironment. CDK1 expression was also correlated with increased infiltration of numerous immune cells, including CD4+ T cells and M1 macrophages. Patients with high CDK1 expression tended to have a poor response to immunotherapy but were sensitive to multiple chemotherapies and targeted drugs. The MDK-NCL and SPP1-CD44 ligand−receptor pairs were markedly activated in the intercellular communication network. CDK1 was an independent prognostic factor for LUAD and improved the ability to predict overall survival when combined with tumor stage.

**Conclusion:**

CDK1 plays an essential role in reshaping the tumor immune microenvironment and might be a prognostic and treatment biomarker in LUAD.

## 1 Introduction

Lung cancer is a prevalent malignancy worldwide (1), and lung adenocarcinoma (LUAD) is the most common histological subtype, accounting for nearly 40% of all lung cancer cases (2). In recent decades, the development of precision medicine has improved the prognosis of LUAD (3). However, most patients experience resistance to targeted treatments, and only a small percentage of patients are sensitive to immunotherapy (4, 5). Overall, clinical outcomes of LUAD patients are still far from satisfactory. New biomarkers that can accurately predict individualized treatment response and improve prognosis in LUAD are urgently needed.

Cyclin-dependent kinases (CDKs) are a set of serine/threonine protein kinases that participate in the cell cycle process (6). With the induction of cyclins, each CDK is activated alternately to drive the completion of the cell cycle in an orderly manner (7). There are 21 genes encoding CDKs and 5 genes encoding CDK-like (CDKL) kinases (8), and these proteins are involved in the regulation of transcription, epigenetic mechanisms, metabolic processes, and the self-renewal of stem cells (7, 9). Due to their crucial role in cell proliferation, CDKs are considered natural targets for antitumor treatment (6).

Preclinical experiments have proven the significant antitumor effect of CDK inhibitors in multiple malignancies (10). Palbociclib, ribociclib, and other CDK4/6 inhibitors have been approved by the FDA for the clinical treatment of breast cancer (11). For other types of solid tumors, including LUAD, pan-CDK inhibitors have not shown the expected results in clinical trials because of a lack of selectivity (12). To solve this problem, it is necessary to recognize the role of each CDK in the pathogenesis of different cancer types as well as their genetic characteristics and their interactions with signaling pathways and the tumor microenvironment (TME).

In this study, we explored the transcriptomes of 26 CDK family genes in LUAD and identified CDK1 as a candidate therapeutic target and prognostic marker. In addition, we verified the important role of CDK1 in cell proliferation and found that cells with high CDK1 expression interacted with multiple signaling pathways to reshape the tumor immune microenvironment. Finally, we proposed rational suggestions for the treatment of groups of patients with different CDK1 expression levels and constructed a nomogram to optimize the prediction of prognosis for LUAD patients.

## 2 Materials and methods

### 2.1 Datasets and samples

The RNA sequencing (n = 492), somatic mutation (n= 482), copy number variation (CNV) (n = 480), and corresponding clinical data used in this study were downloaded from The Cancer Genome Atlas (TCGA) database (https://portal.gdc.cancer.gov). In addition, three independent cohorts from GEO datasets (https://www.ncbi.nlm.nih.gov/) were used for validation: GSE31210 (n = 226), GSE68465 (n = 439), and GSE72094 (n = 393). Only patients with complete follow-up and tumor stage information were enrolled. **Table 1** shows the baseline clinical characteristics of patients with LUAD in the TCGA and GEO datasets.

**Table 1:** Clinical characteristics of patients with LUAD from multiple cohorts.

Characteristics	TCGA	GSE31210	GSE68465	GSE72094
	N=492	N=226	N=439	N=393
Age (years)				
Median	66	64	65	70
Range	33-88	30-76	33-87	38-89
NA	10	0	0	0
Sex				
Male	227	105	221	174
Female	265	121	218	219
Smoking				
Yes	408	111	298	298
No	71	115	48	30
NA	13	0	93	65
TNM stage				
I	267	168	276	254
II	118	58	95	67
III	81	/	68	57
IV	26	/	/	15
OS status				
Alive	313	191	204	282
Dead	179	35	235	111

NA, Not available; OS, Overall survival.

### 2.2 Expression landscape, prognostic value, and gene alterations of CDK family genes

Mutation waterfall plots of CDK family genes were generated using the “maftools” package based on the somatic mutation data from the TCGA database. The copy number alteration analysis results were visualized in R using the “barplot” function. Due to insufficient normal lung tissue specimens in the TCGA database, we compared the mRNA expression of CDK family genes in tumor and normal tissues *via* the GEPIA (http://gepia.cancerpku.cn) database, which integrates RNA-seq data from the TCGA and GTEx databases. The associations between the expression of CDK genes and the outcomes of LUAD patients were analyzed using univariate Cox regression analysis, and the results were visualized by the “foresplot” package.

### 2.3 Pancancer analysis of CDK1 expression and its associations with clinical characteristics

The mRNA expression data of CDK1 for the TCGA pancancer analysis was obtained from Gene Set Cancer Analysis (GSCA) (http://bioinfo.life.hust.edu.cn/GSCA/#/). The protein expression data of CDK1 and the correlations with mRNA expression in tumor and normal tissues were obtained from the cProSite web (https://cprosite.ccr.cancer.gov/#/). The prognostic value of CDK1 in across cancers was analyzed using GEPIA2.0 (http://gepia2.cancer-pku.cn/#index). The correlation between CDK1 expression and clinicopathological characteristics of patients with LUAD was analyzed using ANOVA or Wilcoxon test and visualized by the “ggplot2” package.

### 2.4 Functional enrichment analysis and gene set variation analysis

Genes related to CDK1 were identified by Pearson correlation analysis using the TCGA and three GSE datasets. Overlapping genes were identified with the criteria Cor >0.5 and p <0.05 and uploaded to the Database for Annotation, Visualization, and Integrated Discovery (DAVID) (https://david.ncifcrf.gov/). Gene Oncology (GO) function enrichment analysis and Kyoto Encyclopedia of Genes and Genomes (KEGG) analysis results were generated. The biological function gene list was downloaded from the GSEA website (https://www.gsea-msigdb.org). We calculated the functional enrichment score of each LUAD sample and generated a heatmap using the “pheatmap” package. The association between CDK1 expression and the enrichment score was assessed using Pearson correlation analysis.

### 2.5 Protein–protein interaction network analysis

The CDK1 coexpressed gene set was input into the STRING (https://cn.string-db.org/) database to build a PPI network. The Mcode plugin of Cytoscape was used to identify the most connected subnetworks under the default parameters. The biological functions of those highly interconnected genes were further visualized by the Cluego plugin.

### 2.6 Gene alteration analysis

cBioPortal (https://www.cbioportal.org/) was used to identify modifications of the CDK1 gene across cancers, and specifically in LUAD, based on TCGA data. Mutation waterfall plots and the most enriched pathway of the high and low CDK1 expression groups were identified by the “maftools” package. The DNMIVD (http://www.unimd.org/dnmivd/) database was used to compare the methylation level of CDK1 between tumor and normal samples. The association between CNVs of CDK1 and immune cell infiltration was analyzed by the TIMER (https://cistrome.shinyapps.io/timer/) database.

### 2.7 Immune infiltration analysis and immunotherapeutic response prediction

The online web tool CIBERSORT (https://cibersort.stanford.edu/) was employed to determine the immune cell infiltration levels of each sample by uploading the gene expression matrix of LUAD. Furthermore, the associations between CDK1 and immunomodulators, major histocompatibility complex (MHC) molecules, lymphocytes, chemokines, and receptors were analyzed using TISIDB (http://cis.hku.hk/TISIDB/index.php), which is an integrated repository for examining interactions between tumors and the immune system. Tumor mutation burden (TMB) values were calculated by the “maftools” package. The tumor immune dysfunction and exclusion (TIDE) score was obtained from the TIDE website (http://tide.dfci.harvard.edu) by uploading the normalized gene expression matrices. The estimated values of different CDK1 expression groups were compared by the Wilcoxon test.

### 2.8 Drug sensitivity analysis

The chemotherapeutic response of TCGA, GSE31210, GSE68465, and GSE72094 patients was evaluated based on drug sensitivity data from the Cancer Genome Project (CGP) database. The half-maximal inhibitory concentrations (IC50) of 251 antitumor drugs was estimated according to the gene expression matrix of each LUAD patient using the “pRRophetic” package. The sensitivity of drugs was compared between low and high CDK1 expression groups. Furthermore, we calculated the correlation coefficient between drug sensitivity and the expression of CDK1 by the Spearman correlation test.

### 2.9 Single-cell sequencing

Single-cell RNA sequencing (scRNA-seq) data of eighteen LUAD samples were obtained from the GEO database (GSE148071) and analyzed by the “Seurat” package. We removed cells that had a percentage of mitochondrial genes >20%. Cells with less than 200 or over 5000 expressed genes were also discarded. The “FindClusters” function was utilized to identify cell clusters. Major cell types were identified by a canonical marker expressed by each cluster: endothelial cells (PECAM1) (13), epithelial cells (SNTN) (13), fibroblasts (COL1A2) (13), T cells (CD2) (13), B cells (IGKC) (14), neutrophils (CSF3R) (13), dendritic cells (CD83) (15), mast cells (TPSAB1) (13), cancer cells (EPCAM) (13), and macrophages (APOC1) (16) (**Figure S5C**). The differentially expressed genes were detected using the “FindMarkers” function. Intercellular communication analysis was performed by the “Cellchat” package.

### 2.10 Prognosis analysis and nomogram construction

Kaplan–Meier curves were assessed using the log-rank test and plotted by the “ggplot2” package. Univariate and multivariate Cox regression analyses were performed using the “survival” package to identify the independent prognostic factors of LUAD. The “rms” package was applied to establish a nomogram and plot the calibration chart. Receiver operating characteristic (ROC) curves were generated by the “survivalROC” package and used to assess predictive ability.

### 2.11 Statistical analysis

The R programming language (Version 4.2.2) was used for data analysis, and p < 0.05 represented statistical significance.

## 3 Results

### 3.1 Expression landscape of CDK family genes in LUAD

**Figure 1:**
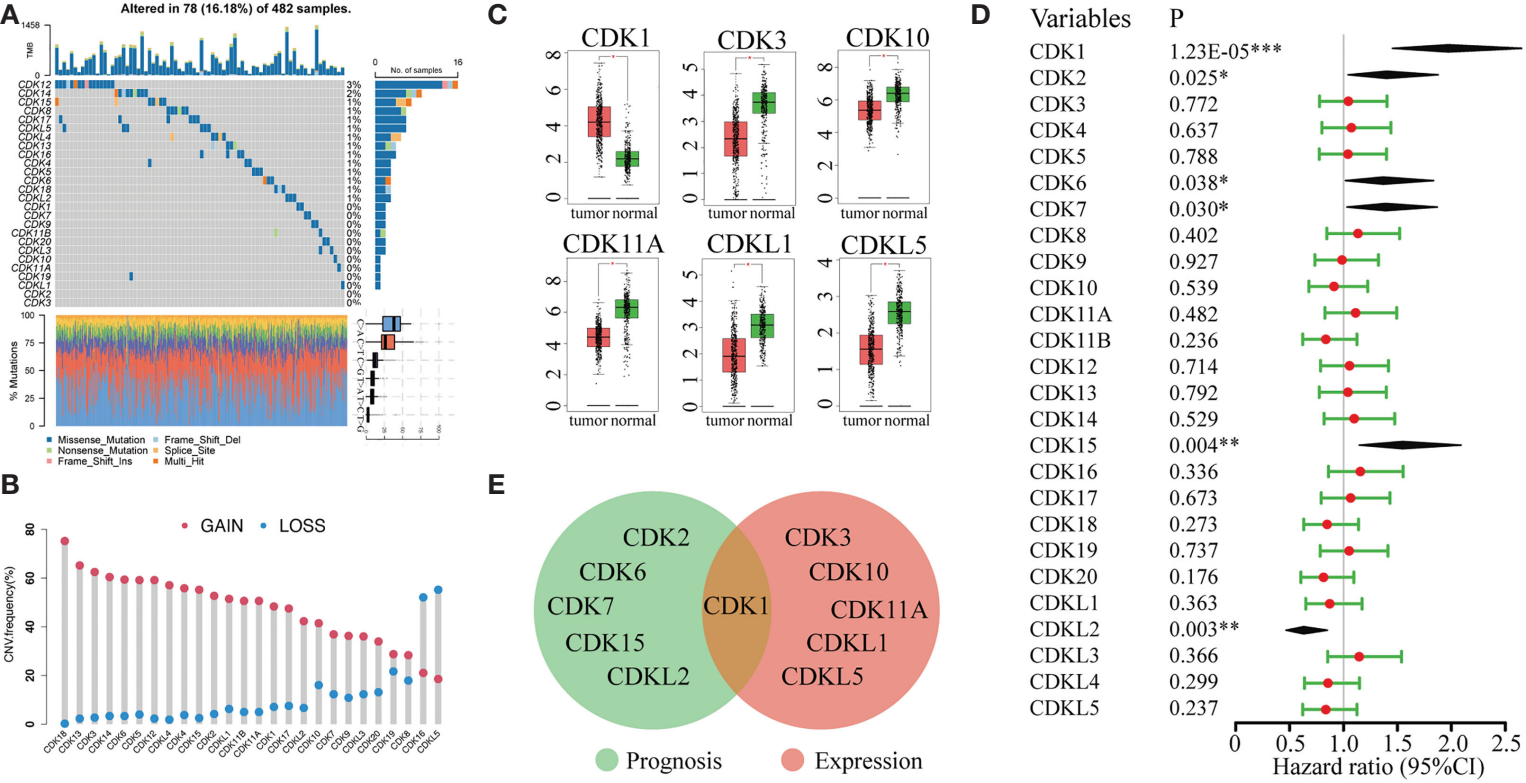
Genetic variation, mRNA expression, and prognostic significance analyses of CDK family genes. **(A)** Mutation frequencies of CDK genes in 482 patients in the LUAD TCGA dataset. Each column represents one patient, the bar on the top represents the tumor mutation burden, and the numbers on the right represent the mutation frequency of each CDK gene. **(B)** CNV frequencies of CDK genes in LUAD. The blue dots represent the frequency of copy number deletion, the red dots represent the frequency of copy number amplification, and the height of the columns represents the change frequency. **(C)** Differentially expressed genes of CDK family members in tumor tissue (red) and normal lung tissue (green). **(D)** Univariate Cox regression analysis of CDK family genes in LUAD. **(E)** Venn analysis of genes that were both differentially expressed between tumor and normal lung tissue and associated with prognosis in LUAD. (*p < 0.05, **p < 0.01, ***p < 0.001).

The frequency of somatic mutations in CDK family genes was 16.18% (78/482 samples), and CDK12 and CDK14 were the most frequently mutated genes in LUAD (**Figure 1A**). All CDK family genes exhibited varying degrees of CNV. Copy number amplification was frequent for CDK18 and CD13, while copy number deletion was prevalent for CDKL5 and CDK16 (**Figure 1B**). Additionally, the mRNA expression of CDK genes in LUAD and normal lung tissues was evaluated. CDK1 was highly enriched in tumors compared to normal lung samples. Furthermore, CDK3, CDK10, CD11A, CDKL1, and CDKL5 were highly expressed in normal samples (**Figures 1C**).

We also evaluated the relationship between the expression levels of CDK family genes and patient prognosis in the TCGA database. High expression of CDKL2 was associated with long overall survival (OS) in LUAD patients. In contrast, high expression of CDK1, CDK2, CDK6, CDK7, and CDK15 was correlated with worse prognosis (**Figure 1D**). However, among all genes associated with LUAD prognosis, only CDK1 was differentially expressed in tumor and normal lung tissues (**Figure 1E**), indicating that CDK1 might be a valuable biomarker for prognostic prediction and targeted therapy in LUAD.

### 3.2 High CDK1 expression was found across cancers and correlated with poor prognosis

**Figure 2:**
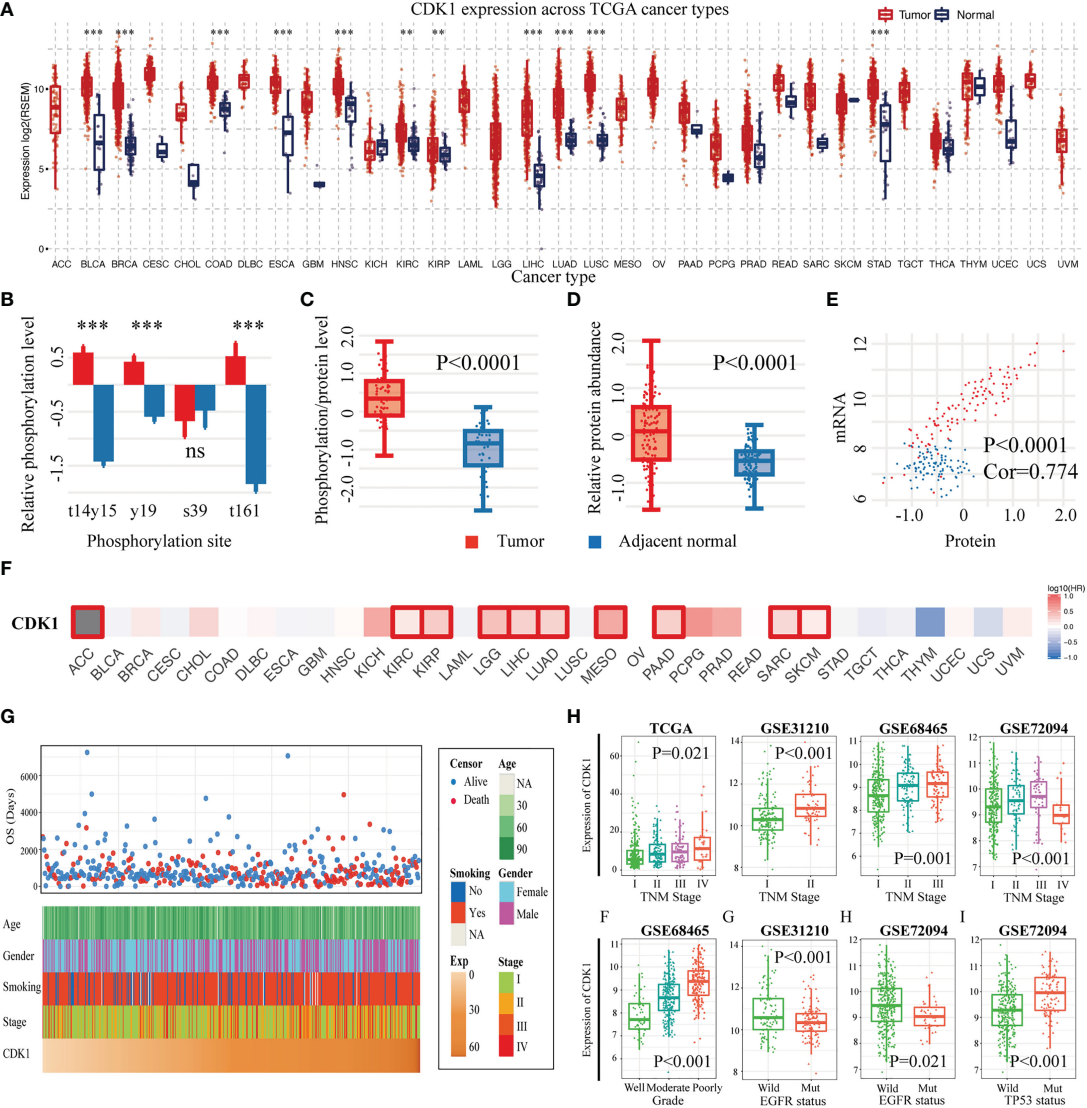
CDK1 expression levels, prognostic value and associations with clinicopathological characteristics across cancers and specifically in LUAD. **(A)** CDK1 expression levels across cancers according to TCGA data. **(B)** Relative phosphorylation level of different CDK1 phosphorylation sites in LUAD samples in comparison to that in healthy tissue samples from the TCGA database. **(C)** Phosphorylated CDK1 protein and **(D)** total CDK1 protein abundance in tumor and normal lung tissues from the TCGA database. **(E)** Correlation between the mRNA expression and the protein abundance of CDK1. **(F)** Relationship between the expression of CDK1 and overall survival across cancers based on TCGA data. **(G)** CDK1 relationships with clinical features of patients with LUAD based on TCGA data. **(H)** CDK1 was significantly increased in LUAD samples with a high degree of malignancy, as indicated by factors such as advanced TNM stage, poor differentiation, wild-type EGFR, and tp53 mutation. (*p < 0.05, **p < 0.01, ***p < 0.001).

With the GSCA database, we found that CDK1 was expressed at high levels in the vast majority of malignancies, including LUAD (**Figure 2A**). In addition, we verified the expression of CDK1 at the protein level in LUAD and normal tissues with the CTPAC database. The phosphorylation of CDK1 at the t14, y15, y19, and t161 sites was significantly increased in LUAD compared to adjacent normal tissues (**Figure 2B**). The levels of both phosphorylated and total CDK1 protein were high in LUAD (**Figure 2C, D**), and the total CDK1 protein level correlated well with the corresponding mRNA expression level (**Figure 2E**). The prognostic value of CDK1 across cancers was analyzed using GEPIA2.0, and the results indicated that patients with various cancers, such as kidney renal clear cell carcinoma, brain lower grade glioma, liver hepatocellular carcinoma, and LUAD, with high CDK1 expression tended to have shorter OS (**Figure 2F**).

### 3.3 Expression of CDK1 was enriched in LUAD cases with a higher degree of malignancy

The expression of CDK1 in LUAD was found to be related to different clinical traits. **Figure 2G** shows the relationships of CDK1 expression with age, sex, smoking history, tumor stage, and overall survival time for LUAD patients in the TCGA database. CDK1 was highly expressed in LUAD cases with advanced tumor-node-metastasis (TNM) stage, which was consistent with the results from the GSE31210, GSE68465, and GSE72094 datasets. (**Figure 2H**). Poorly differentiated tumors showed high expression of CDK1 in the GSE68465 dataset (**Figure 2H**). Moreover, CDK1 tended to be highly expressed in tumors with wild-type epidermal growth factor receptor (EGFR) and TP53 mutation (**Figure 2H**). Overall, these results suggested that more malignant LUAD tumors, such as those with advanced TNM stage, poor differentiation, and tp53 mutation, have higher expression of CDK1.

### 3.4 CDK1 is involved in multiple biological processes including immune cell regulation

**Figure 3:**
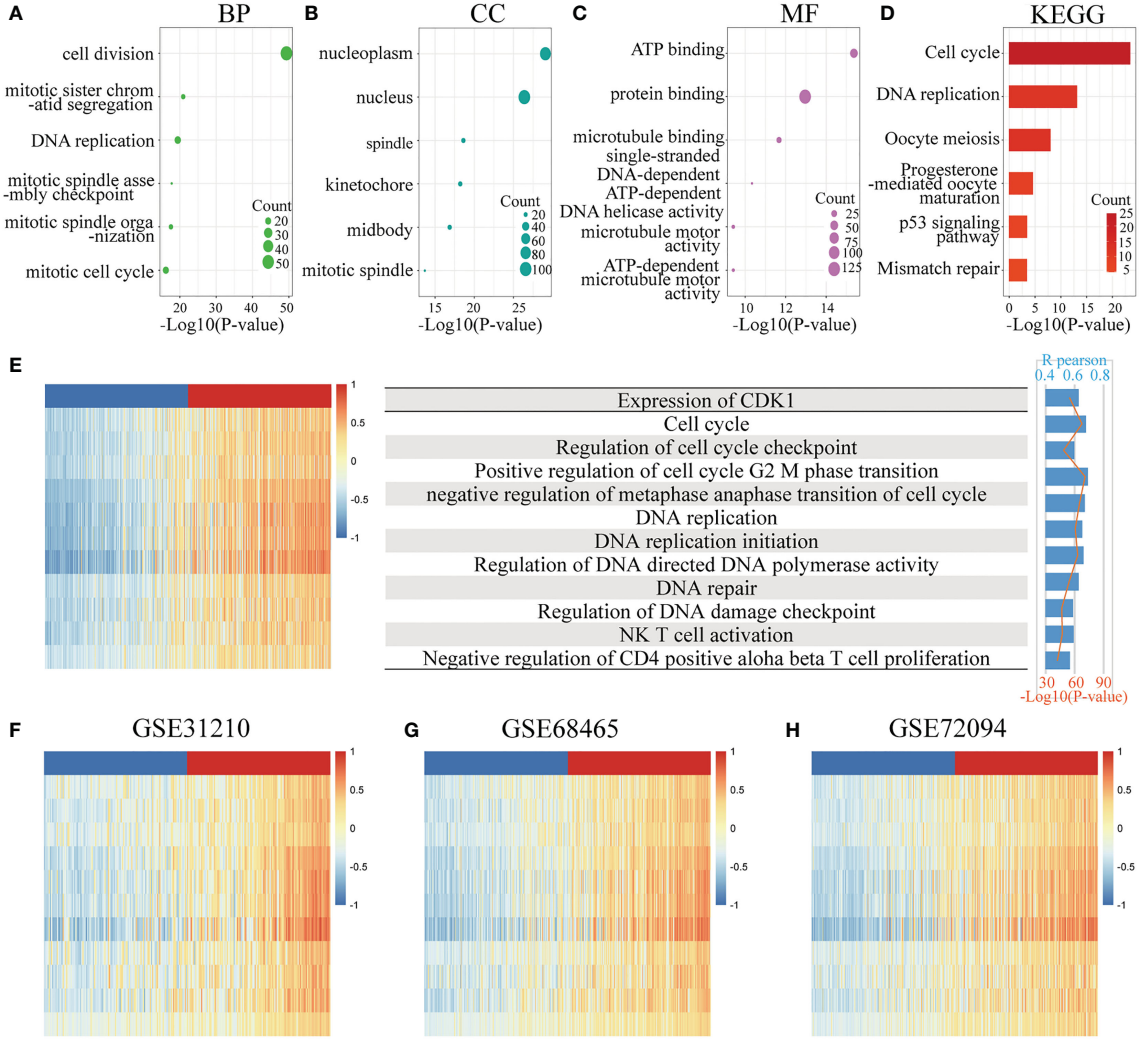
Functional enrichment analysis. **(A–C)** Top 6 GO analysis terms of CDK1 coexpressed genes, including biological process (BP), cellular component (CC), and molecular function (MF) terms. **(D)** Top 6 KEGG analysis terms of CDK1 coexpressed genes. **(E–H)** Analysis of the correlation between CDK1 expression and functional enrichment scores of each patient in the TCGA, GSE31210, GSE68465, and GSE72094 datasets.

To clarify the biological functions of CDK1 in LUAD, the genes strongly related to CDK1 were identified by Pearson correlation analysis (R > 0.5, p<0.05) in four dependent datasets (TCGA, GSE31210, GSE68465, and GSE72094). Overall, 136 genes overlapped among the CDK1-associated genes in the above four datasets (**Figure S2A**). GO and KEGG analyses of the coexpressed gene sets were conducted. CDK1 was found to be associated with the following biological processes: cell division, mitotic sister chromatid segregation, and DNA replication (**Figure 3A**). In addition, the cellular components most related to CDK1 were found to be the nucleoplasm and nucleus (**Figure 3B**). The top molecular function term related to CDK1 was ATP binding (**Figure 3C**). The signaling pathway terms with the strongest relationship with CDK1 were the cell cycle, DNA replication, and oocyte meiosis (**Figure 3D**). These results indicated that CDK1 plays an essential role in cell proliferation and energy metabolism in LUAD.

GSVA revealed that CDK1 expression had a positive correlation with the enrichment score of multiple biological functions, such as positive regulation of cell cycle G2/M phase transition, regulation of DNA-directed DNA polymerase activity, and regulation of DNA damage checkpoints. In addition, CDK1 expression was positively related to natural killer (NK) T-cell activation and negative regulation of CD4-positive alpha beta T-cell proliferation, indicating that CDK1 also plays a role in regulating immune cells (**Figures 3E–H**).

### 3.5 CDK1 coexpressed genes were mostly enriched in tumor proliferation pathways in the PPI network

The “Mcode” plug-in identified seven highly interacting subsets of CDK1 coexpressed gene sets, with a total of 83 nodes and 3880 edges (**Figure S2B**). These closely interconnected gene clusters were most often involved in the mitotic cell cycle, DNA replication, homologous recombination, and the p53 signaling pathway. **Figures S2C–F** shows the relationships of these biological processes. Nodes are representative of enriched pathways. The interconnecting lines of nodes indicate the genes shared between the connected nodes. The direction of the lines indicates the relationship between these biological processes in oncology.

### 3.6 CDK1 upregulation in LUAD is mainly contributed by DNA copy number gain

An analysis of the TCGA database was carried out to identify genetic modifications of CDK1. The alteration frequency of CDK1 in various cancers did not vary greatly, and approximately 1.2% of patients with LUAD had genetic abnormalities, with the main types being “mutation” and “amplification” (**Figure S3A**). We identified the top 20 most mutated genes in different CDK1-expressing subgroups. High expression of CDK1 most often occurred with mutations in the TP53 (66%), TTN (53%), and CSMD3 (46%) genes (**Figure S3B**), while low expression of CDK often co-occurred with MUC16 (35%), TTN (32%), and TP53 (31%) mutations (**Figure S3C**). Overall, gene mutations occurred more frequently in the high CDK1 expression cohort than in the low expression cohort. RTK-RAS, WNT, and NOTCH were the most affected pathways associated with the mutated genes in the low and high expression cohorts (**Figures S3D, E**). The cBioPortal database was used to assess the effects of CNV on the mRNA expression of CDK1 in LUAD. We found that the mRNA expression in samples with “gain” abnormalities of CDK1 was significantly higher than that in samples with “shallow deletion” and “diploid” abnormalities of CDK1 in LUAD (**Figure S3F**). Regarding DNA methylation, we discovered that there was no apparent difference in the methylation level of CDK1 between tumor and normal samples (**Figure S3G**), indicating that the promoter methylation of CDK1 may not cause changes in the mRNA level. In addition, “arm-level gain” CNVs had an inverse association with the infiltration of immune cells, including B cells, CD4+ T cells, and dendritic cells (**Figure S3H**).

### 3.7 CDK1 reshapes the immune microenvironment in LUAD

**Figure 4:**
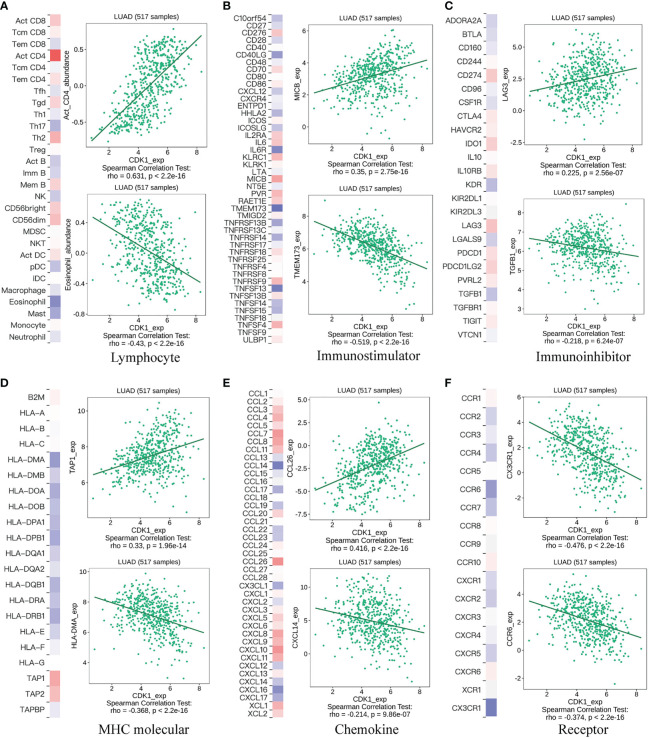
Correlation of CDK1 gene expression with lymphocytes, immunomodulators, and chemokines. **(A)** Relationship between CDK1 and lymphocytes. **(B)** Relationship between CDK1 and immunostimulators. **(C)** Relationship between CDK1 and inhibitory immune molecules. **(D)** Relationship between CDK1 and MHC molecules. **(E)** Relationship between CDK1 and chemokines. **(F)** Relationship between CDK1 and chemokine receptors.

The GSVA results revealed that CDK1 also plays a role in immune regulation, especially in T cells. Tumor-infiltrating lymphocytes have been reported as a predictor of disease progression and to be related to the efficacy of immunotherapy in non-small cell lung cancer (NSCLC) patients (17, 18). Here, we next performed a comprehensive analysis to investigate the relationship between CDK1 expression and tumor immune microenvironment factors, including lymphocytes, checkpoints, MHC molecules, chemokines and receptors. **Figure 4** shows the molecules with the highest associations: lymphocytes: activated CD4+ T-cells (r=0.631, p<2.2e-16), eosinophils (r=-0.43, p<2.2e-16); immunostimulators: MICB (r=0.35, p=2.75e-16) and TMEM173 (r=-0.519, p<2.2e-16); inhibitory immune molecules: LAG-3 (r=0.225, p=2.56e-7), TGFB1 (r=-0.218, p=6.24e-7); MHC molecules: TAP1 (r=0.33, p=1.96e-14), HLA-DMA (r=-0.368, p<2.2e-16); chemokines: CCL26 (r=0.416 p<2.2e-16), CXCL14 (r=-0.214 p=9.86e-7); and chemokine receptors: CX3CR1 (r=-0.476, p<2.2e-16), CCR6 (r=-0.374, p<2.2e-16). Those results were also validated in the GSE31210, GSE68465, and GSE72094 datasets (**Figures S4A-C**).

**Figure 5:**
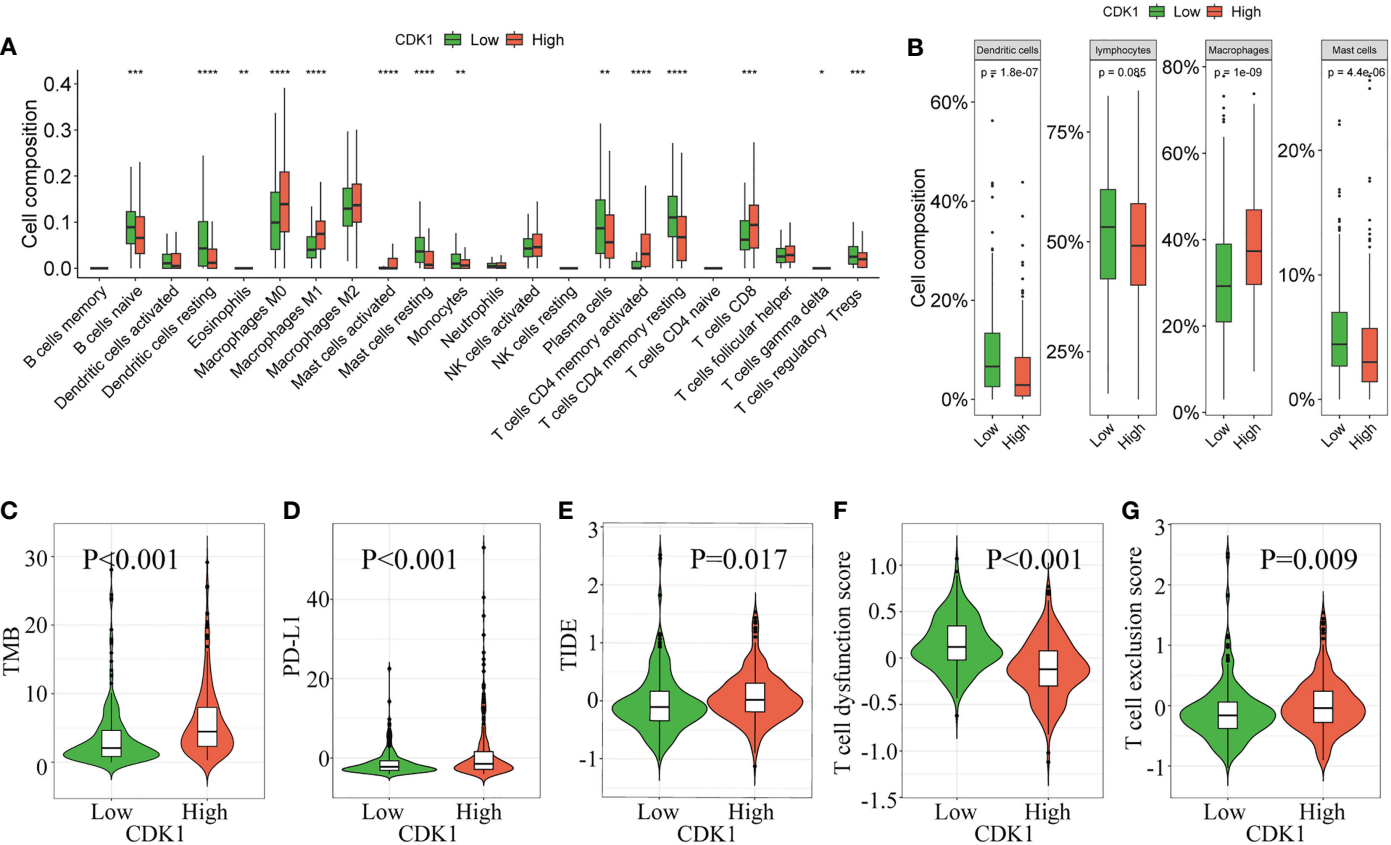
Correlation of CDK1 expression with immune cell infiltration and immunotherapy response in the TCGA cohort. The immune cell composition of groups of patients with LUAD with different CDK1 expression, including 22 immune cell types **(A)** and 4 aggregated immune cell types **(B)**, is shown. **(C–G)** Comparison of immunotherapy response biomarkers, including tumor mutation burden (TMB), PD-L1 score, tumor immune dysfunction and exclusion (TIDE) score, T-cell dysfunction score and T-cell exclusion score, between the CDK1 high and low expression groups in the TCGA cohort. (*p < 0.05, **p < 0.01, ***p < 0.001, ****p < 0.0001).

The difference in immune cell infiltration between different CDK1 expression groups was further validated using the CIBERSORT algorithm in the TCGA database. M0 and M1 macrophages, activated mast cells, activated CD4+ memory T cells, and CD8+ T cells were significantly upregulated in the cohort with high CDK1 expression, and resting dendritic cells, resting mast cells, monocytes, and resting CD4+ memory T cells were significantly downregulated (**Figure 5A**). Overall, expression of CDK1 in LUAD was positively associated with the levels of infiltrating macrophages and negatively correlated with the levels of infiltrating dendritic cells and mast cells (**Figure 5B**). These findings were consistent with the results from the GSE31210, GSE68465, and GSE72094 datasets (**Figures S4D, F, H**).

### 3.8 High CDK1 expression indicates a poor response to immunotherapy

CDK1 had a positive association with TMB and PD-L1 (**Figures 5C, D**), which indicated that patients with high expression of CDK1 had an enhanced ability to recruit immune cells to identify tumors. However, patients with high CDK1 expression also had higher TIDE scores and T-cell dysfunction scores and lower T-cell exclusion scores (**Figures 5E–G**), suggesting a higher possibility of escape from immune surveillance and poorer response to immunotherapy. Previous studies have found that compared with conventional biomarkers, such as TMB and PD-L1, the TIDE score had a superior ability to evaluate the efficacy of anti-PD1 and anti-CTLA4 therapy (19). Overall, immunotherapy is more likely to benefit patients with low CDK1 expression, and CDK1 may serve as a biomarker to identify candidates for immunotherapy among patients with LUAD. Those results were also validated in the three validation cohorts (**Figures S4E, G, I**).

### 3.9 CDK1 expression is associated with the efficacy of multiple antitumor drugs

**Figure 6:**
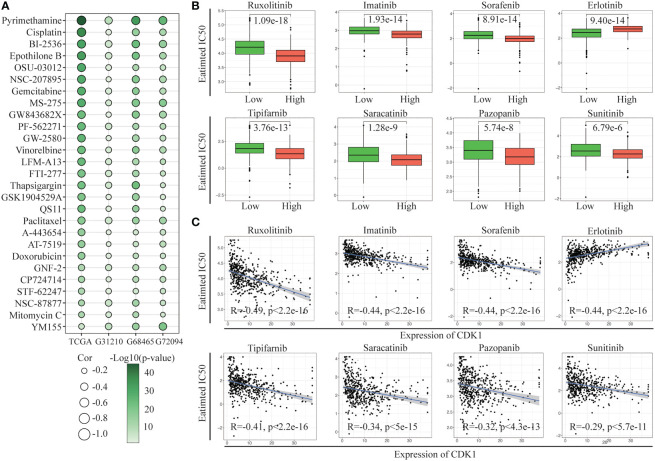
Evaluation of drug sensitivity. **(A)** Correlation of CDK1 expression with the estimated IC50 values of 27 drugs in the TCGA, GSE31210, GSE68465, and GSE72094 cohorts. **(B)** The comparisons in IC50 value of 8 targeted drugs between the CDK1 high and low expression groups in the TCGA cohort. **(C)** Correlation of CDK1 expression with the estimated IC50 values of 8 targeted drugs in the TCGA cohort.

Further exploration of the IC50 levels of chemotherapy drugs was conducted among patients with different CDK1 expression levels from the TCGA and GSE31210, GSE68465, and GSE72094 cohorts. Twenty-seven overlapping drugs were identified with the criteria p< 0.05 and correlation coefficient < -0.2 (**Figure 6A**). Patients with high CDK1 expression tended to have higher sensitivity to anticancer drugs, especially pyrimethamine, cisplatin, BI-2536, epothilone B, and OSU-03012, than those with low CDK1 expression.

We also investigated the sensitivity of the two groups to multiple targeted drugs. Patients with high CDK1 expression had lower IC50 values for most targeted drugs, such as ruxolitinib, imatinib, sorafenib, and tipifarnib, than patients with low expression, indicating that targeted therapy might have better efficacy in patients with high expression of CDK1 (**Figure 6B**). In addition, patients with low CDK1 expression were more sensitive to erlotinib. The IC50 values of all targeted drugs were significantly correlated with the expression of CDK1 (**Figure 6C**).

### 3.10 CDK1 expression in cells is correlated with MDK/NCL and SPP1/CD44 ligand–reception pairing in LUAD

**Figure 7:**
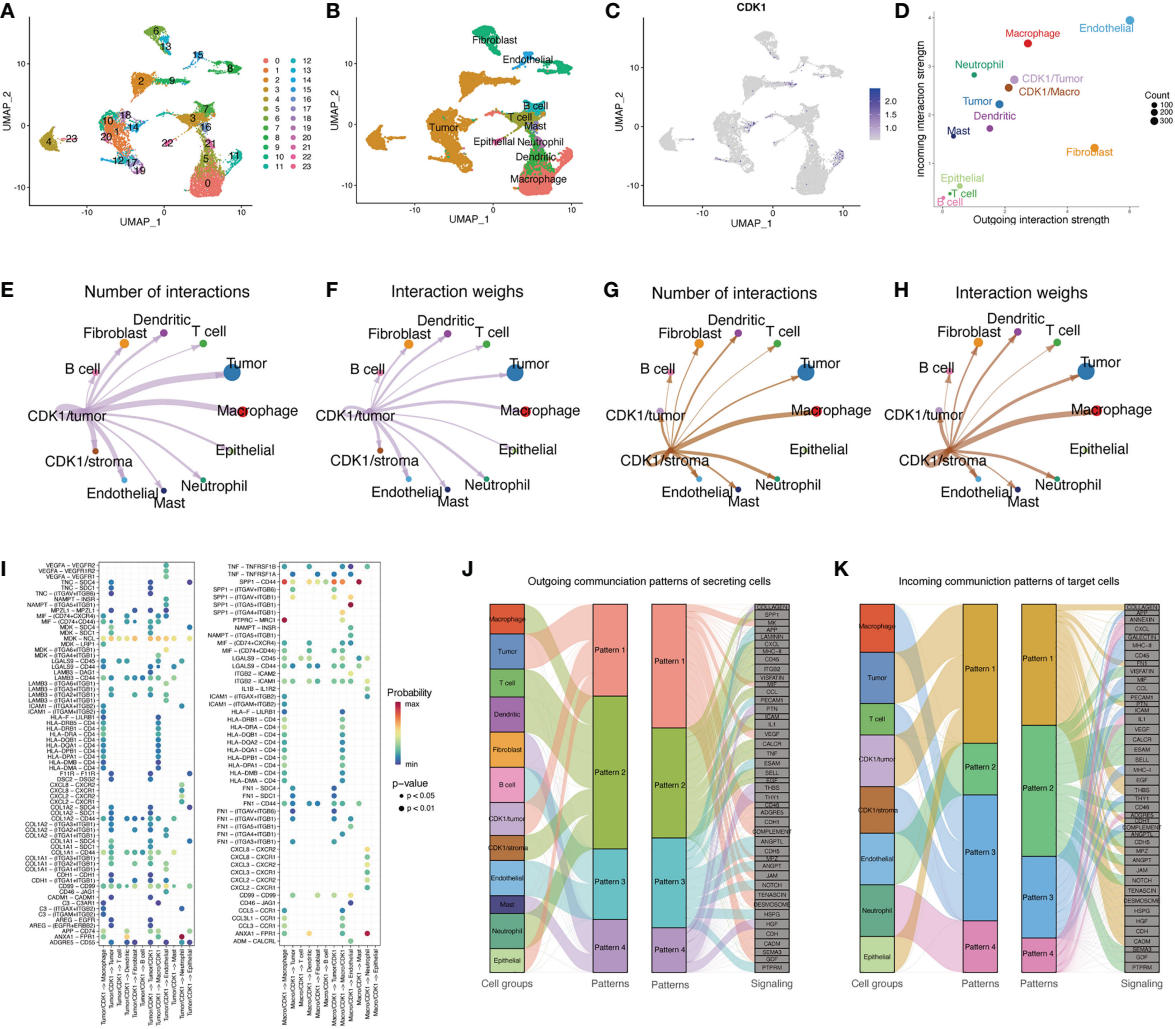
Single-cell analysis of CDK1. **(A)** UMAP plot from 18 patients colored by 23 clusters. **(B)** UMAP plot colored by 10 major cell types. **(C)** CDK1 expression in different cells. **(D)** The interaction weight of 12 cell types acting as senders or receivers of signals in the communication network. **(E, F)** Cell–cell communication relationships between 12 main cell types and CDK1/tumor cluster cells. **(G, H)** Cell–cell communication relationships between 12 main cell types and CDK1/stroma cluster cells. **(I)** All the significant ligand−receptor pairs that contributed to signaling from CDK1/tumor or CDK1 stroma cluster cells to other cell types. The dot color and size represent the calculated communication probability and p values, respectively. **(J)** Inferred outgoing communication patterns of secreting cells, including the correlations between the inferred latent patterns and cell groups, as well as signaling pathways. The thickness of the line indicates the contribution of the cell group or signaling pathway to each latent pattern. **(K)** Inferred incoming communication patterns of target cells.

By analyzing the scRNA-seq data of 18 LUAD samples, we obtained 23 distinct clusters (**Figure 7A**) and identified 10 major cell types (**Figure 7B**), including T cells, B cells, cancer cells, dendritic cells, epithelial cells, endothelial cells, fibroblasts, macrophages, mast cells, and neutrophils. CDK1 was mostly expressed in the 4, 9, 11 and 23 clusters, which were primarily tumor cells and macrophages (**Figure 7C**). Clusters with high CDK1 expression exhibited varying degrees of similarity in transcriptome signature and showed high coexpression of TOP2A, ASPM, CEP55 and NUSAP1, which indicated high proliferation of the cells (**Figure S5B**). In addition, we constructed an intercellular communication network to show the interactions of ligand−receptor pairs between different cell clusters. The ligand served as the sender, and the receptor served as the receiver. In the LUAD tissues, endothelial cells, fibroblasts, and macrophages showed the strongest interactions with other cells, while lymphocytes and mast cells exhibited relatively weak intercellular relationships (**Figure S5D**). According to the cell types in which CDK1 was expressed, we classified the CDK1-expressing clusters into two subgroups, the CDK1/tumor cluster and the CDK1/stroma cluster.

**Figure 7D** shows the different roles of each cell type in the network. Endothelial cells and fibroblasts mainly acted as signal senders, while macrophages, CDK1/stromal cluster cells, tumor cells, and CDK1/tumor cluster cells mainly acted as receivers. The CDK1/stroma and CDK1/tumor clusters both had varying degrees of intercellular communication with other cell types in the communication network, especially tumor cells and macrophages (**Figures 7E–H**). **Figure 7I** shows the significant ligand−receptor pairs in the communication network of different cell groups. The interaction between CDK1/tumor cluster cells and other cell types mainly occurred through the MDK/NCL pair, and CDK1/stoma cluster cells mainly interacted with other cells through the SPP1/CD44 and SPP1/(ITGAV+ITGB1) pairs. **Figures 7J, K** shows the relationships between multiple cell groups and their communication patterns that drive certain signaling pathways. Macrophages, T cells, dendritic cells, CDK1/stroma cluster cells, and neutrophils output their signals mainly through pattern #2, which represents signaling pathways such as the SPP1, CXCL, MCH-II, and TNF pathways. Tumor cells, CDK1/tumor cluster cells, and epithelial cells all output signals *via* pattern #1, including factors such as MK, MIF, VEGF, and CD46. On the other hand, incoming signaling of CDK1/stroma cluster cells, macrophages, and T cells was characterized by pattern #3, driven by the MHC-II, CD45, and CCL pathways. Interestingly, both the incoming and outgoing patterns of CDK1/tumor cells were similar to those of tumor and epithelial cells. These findings indicate that cells with CDK1 expression may activate multiple pathways simultaneously with other cell types through overlapping signaling networks.

### 3.11 CDK1 can independently predict the OS of patients with LUAD

**Figure 8:**
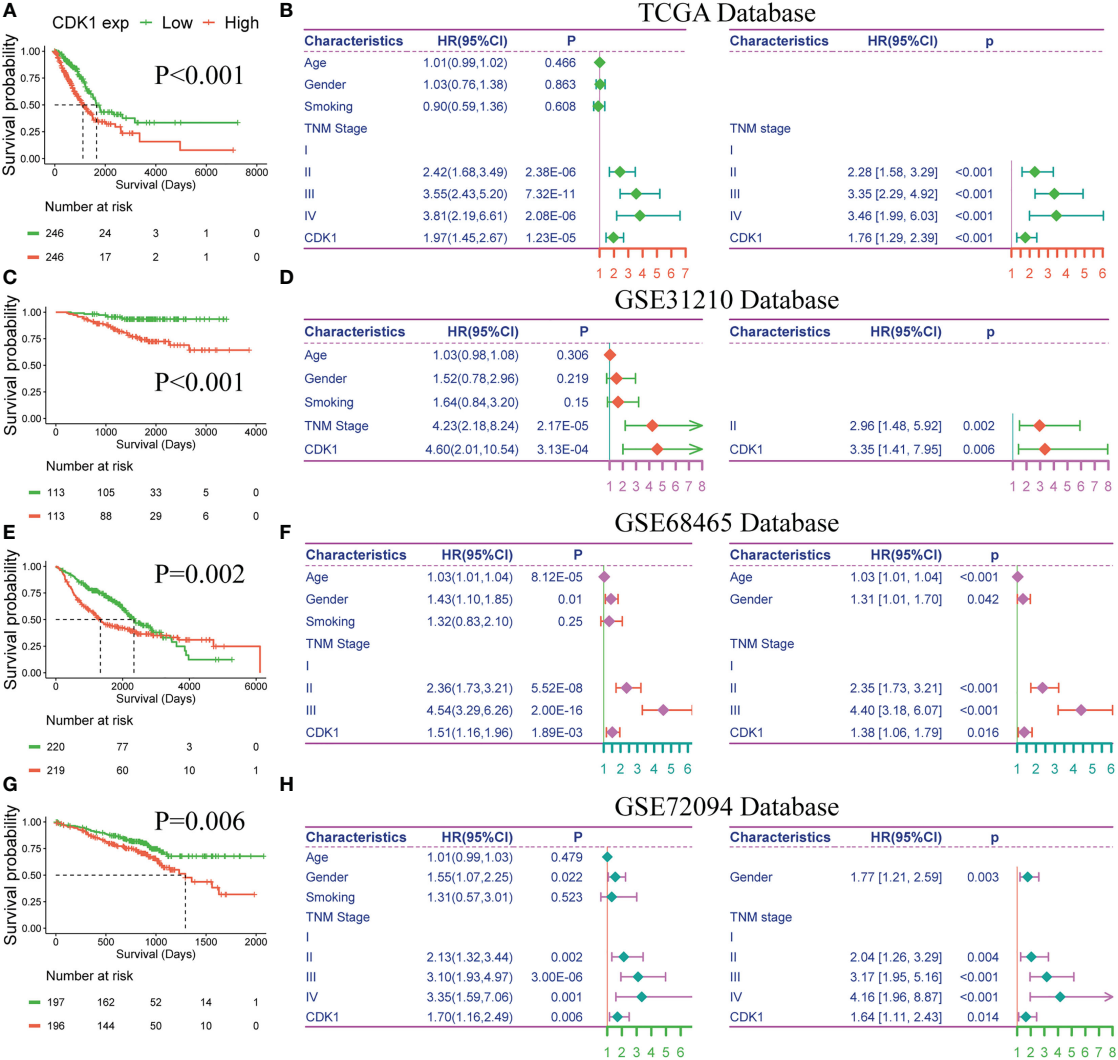
Prognostic value of CDK1 in LUAD. Kaplan−Meier analysis of CDK1 expression in the TCGA **(A)**, GSE31210 **(C)**, GSE68465 **(E)**, and GSE72094 **(G)** datasets. Univariate and multivariate Cox regression analyses of prognostic factors for overall survival in the TCGA **(B)**, GSE31210 **(D)**, GSE68465 **(F)**, and GSE72094 **(H)** datasets.

**Figure 9:**
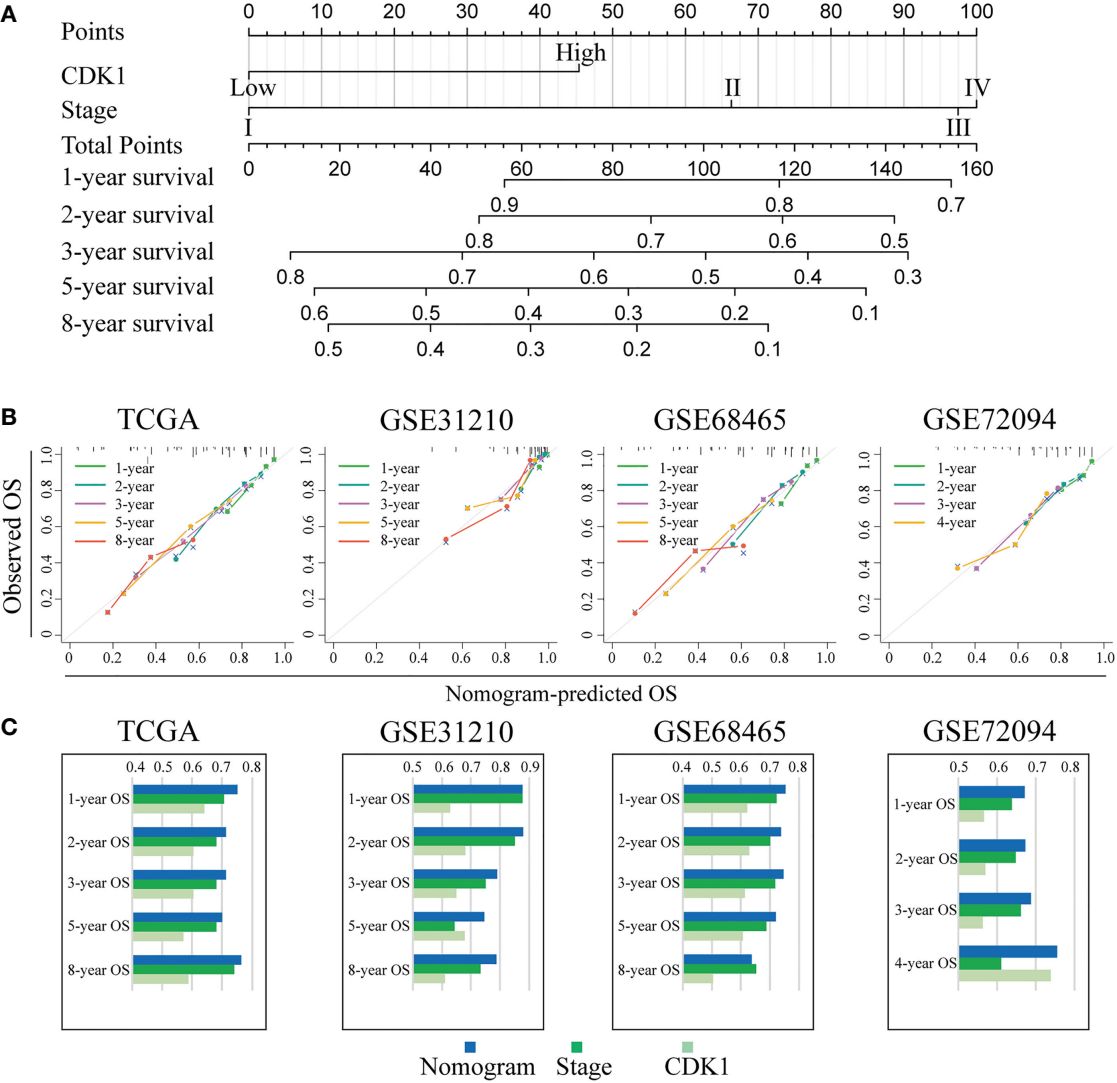
Individualized prediction model for OS in LUAD. **(A)** The 1-, 2-, 3-, 5-, and 8-year OS rates of LUAD patients could be precisely predicted by the nomogram. **(B)** The calibration plots show the comparison between predicted and actual 1-, 2-, 3-, 5-, and 8-year OS in the training and validation groups. **(C)** The predictive ability of the nomogram, TNM stage, and CDK1 expression regarding OS was evaluated based on the area under the ROC curves for 1-, 2-, 3-, 5-, and 8-year OS.

To further explore the prognostic value of CDK1 in LUAD, Kaplan−Meier and Cox regression analyses were performed based on the dataset from TCGA and three independent GEO datasets (GSE31210, GSE68465, and GSE72094). Patients with higher expression of CDK1 had a significantly shorter OS than those with lower CDK1 expression (**Figures 8A, C, E, G**). The Cox regression analyses revealed that CDK1 was a prognostic variable independent of age, sex, smoking status, and TNM stage, which is consistent with the results from GSE31210, GSE68465, and GSE72094 (**Figures 8B, D, F, H**). Using the independent markers of prognosis, we constructed a nomogram to predict 1-, 2-, 3-, 5-, and 8-year OS for LUAD patients (**Figure 9A**). The calibration plots demonstrated that the prediction of the model was well matched with the observed 1-, 2-, 3-, 5-, and 8-year OS rates (**Figure 9B**). The ROC curves indicated that our nomogram had a better ability to discriminate OS than TNM stage in both the training and validation cohorts (**Figure 9C**).

## 4 Discussion

In recent years, a large number of clinical trials of CDK inhibitors have been performed in various cancers (12, 20). The use of CDK4/6 inhibitors optimized the treatment of hormone receptor-positive breast cancer (21, 22). However, these inhibitors did not have comparable clinical benefits in patients with NSCLC (23, 24). The CDK4/6 inhibitor abemaciclib did not show clinical activity, with a PFS of 2.4 months, compared to the 4.2-month PFS of docetaxel, in the second-line treatment of stage IV NSCLC (25). A phase I/II clinical trial of a CDK1 inhibitor plus gemcitabine for pancreatic cancer revealed that this regimen not only inhibited tumor growth but also eliminated resistance to gemcitabine (10). These results suggest that it might be better to explore new targets of the CDK family, such as CDK1, for therapeutic interventions in LUAD (26, 27).

Our study found that the expression of CDK1 in LUAD was significantly higher than that in normal tissues. CDK1 tended to be enriched in tumors with a higher degree of malignancy, as indicated by factors such as more advanced TNM stage, poor differentiation, and tp53 mutation. Patients with high expression of CDK1 had a higher frequency of somatic mutations, implying more instability of the genome and more production of tumor neoantigens, than those with low expression of CDK1 (28). We found that DNA mutation and amplification of CDK1 were rare in LUAD (<1.5%). Furthermore, DNA methylation of CDK1 had little influence on its mRNA expression. The analysis showed that CDK1 promoted tumor cell proliferation mainly through “gain” CNVs, which also negatively regulated the infiltration of multiple immune cells, including B cells, CD4+ T cells, and dendritic cells.

In our study, CDK1 overexpression was associated with mutated TP53 and wild-type EGFR but was not related to K-RAS gene mutations. RAS family genes play an important role in the EGFR signaling pathway and the activated KRAS protein is currently considered to effect the growth, proliferation, and differentiation of tumors (29–31). However, effective therapeutic strategies to target RAS-mutant cancers have proved elusive because of the difficulty in directly inhibiting RAS proteins (32). Using therapeutic combination strategies or harnessing the immune system may be optional approaches for those refractory caners (29). The lack of significant overexpression of CDK1 in patients with EGFR or K-RAS mutations in this study indicated that CDK1 may regulate the growth and proliferation of LUAD tumors *via* a mechanism that does not rely on the EGFR/K-RAS pathway. Besides, KRAS mutation is one of the most common alterations in NSCLC, especially in LUAD (33). From this point of view, CDK1 may have a limited value as a diagnostic biomarker of LUAD and it may be more suitable as a prognostic marker that could have great value and enable clinicians to adapt the individualized therapeutic regimens of LUAD patients.

As a member of the serine/threonine kinase family, CDK1 mainly drives the transition of the cell cycle from interphase to mitosis (34). A previous study revealed that CDK1 is involved in biological processes, including cell division, DNA replication, energy metabolism, and autophagy (35–37), which is consistent with the findings of our study. In addition, CDK1 might also play a role in regulating processes in the tumor microenvironment (TME), such as NK/T-cell activation and CD4-positive alpha beta T-cell proliferation. We found that CDK1 had the most negative correlation with the immunomodulator TMEM173, also known as STING, which plays a crucial role in regulating various functions, including apoptosis, necroptosis, and immunogenic cell death (38). STING can activate type I interferons and TNF-α to upregulate the antitumor activity of T cells and macrophages (39, 40). In addition, CDK1 had a positive association with LAG-3 and PD-L1 (CD274), which shape an immunosuppressive TME to enable tumors to evade immune monitoring (41). CDK1 was negatively related to most MHC molecules, especially the HLA-D family, which participates in antigen presentation. Deficiency of HLA-D can cause tumor cells to be unable to be detected and eliminated by the immune system (42). As the strongest coexpressed chemokine with CDK1, CCL26 has also been proven to recruit tumor-associated macrophages (TAMs) into the tumor matrix by interacting with CCR31 (43). Regarding chemokine receptors, CDK1 had the strongest correlation with CX3CR1. Basic experiments have shown that knockdown of CX3CR1 inhibits the proliferation and invasion of lung cancer cells in mice by switching TAMs toward M1 polarization (44). Furthermore, we observed higher levels of infiltrating macrophages (M0, M1) in the tumor microenvironment of patients with higher CDK1 expression, suggesting that CDK1 might recruit macrophages by upregulating the CCL26/CCR3 pathway and shift TAMs from the M2 to the M1 phenotype by downregulating CX3CL1/CX3CR1 pathway activity. Overall, patients with high CDK1 expression had increased infiltration of immune-activating cells, including M1 macrophages, activated mast cells, activated CD4 memory cells, and CD8-positive T cells, and decreased infiltration of immunosuppressive cells, such as resting dendritic cells, resting mast cells, and resting CD4+ memory T cells. In addition, changes in multiple transcription factors inhibit the antitumor activity of immune cells, including upregulation of LAG-3 and PD-L1 and downregulation of STING and HLA-D. The relationship between CDK1 expression and the tumor microenvironment is complicated, and the tumor microenvironment reshaped by CDK1 is the result of interactions between multiple immune-associated molecules.

We also predicted the response to immunotherapy in subgroups with different expression of CDK1. Patients with high expression of CDK1 tended to benefit less from immunotherapy despite having increased TMB and PD-L1 values. The possible reasons for this discrepancy may be related to the abnormal activation of multiple CDK1-associated cytokine pathways, suggesting that combination therapy, such anti-PD-L1 and anti-LAG-3 combination therapy or immunotherapy combined with anti-CDK1 therapy or chemotherapy, may be a better choice for this group of patients (45). Pyrimethamine is a lipophilic drug and has been considered an effective treatment for malaria in recent years (46). Due to their inhibition of STAT3 signaling, pyrimethamine are being increasingly used in the treatment of human cancer (47). Activation of STAT3 in NSCLC is related to poor clinical outcomes, and preclinical experiments have also revealed that pyrimethamine inhibit LUAD growth (48, 49). In our study, patients with high CDK1 expression had greatly higher sensitivity to pyrimethamine than those with low CDK1 expression. Patients with high CDK1 expression may have better prognosis with pyrimethamine treatment, though further study to validate this idea is warranted. Overall, the results in this study indicated a new possible choice for the treatment of patients with high expression of CDK1, who rarely benefit from immunotherapy. The molecular mechanism underlying those findings is worth exploring in the future.

Single-cell sequencing revealed that CDK1 is mainly localized on tumor cells and macrophages; however, we could not clearly distinguish which specific type of macrophages contained CDK1. Use of the canonical marker genes CD86 and CXCL10 for M1 macrophages and CD163 and MRC1 for M2 macrophages (13, 50, 51) failed to classify macrophages into two subtypes. However, we found that the MDK-NCL pair was specifically activated in the interactions between the CDK1/tumor cluster cells and other cell types, especially tumor cells, fibroblasts, and CDK1/stroma cluster cells. MDK encodes a secreted growth factor that promotes cell growth, migration, and angiogenesis and improves the stem-like properties of cancer cells (52, 53). It is also involved in the pathogenesis of LUAD and induces immune exhaustion in LUAD (54). Based on the results of our study, CDK1/tumor cluster cells may release MDK to promote the proliferation of tumor cells and mediate the exhaustion of immune cells in an autocrine or paracrine manner. In addition, the SPP1-CD44 pair was significantly activated in the interactions between the CDK1/stroma cluster cells and other cell types, especially macrophages, CDK1/tumor cluster cells, and mast cells. Previous studies revealed that SPP1+ TAMs showed higher M2 signatures and were associated with increased resistance to anti-PD-L1 therapy because they reduce lymphocyte infiltration across cancers (55, 56). Moreover, SPP1+ TAMs were found to be positively related to angiogenesis and tumor metastasis (56, 57). These findings suggest that CDK1-expressing cells may contribute to the suppressive immune microenvironment and promote the proliferation of tumor cells and angiogenesis of tumors by secreting MDK and SPP1 into the TME; these ideas partially explain why patients with LUAD with high CDK1 expression benefit little from immunotherapy and have poor survival. Finally, we constructed a detailed map of ligand−receptor interactions in LUAD to identify gene regulatory networks specific to certain cell types. The CDK1/stroma cluster cells, macrophages, and T cells shared a unique communication pattern, and the CDK1/tumor cluster cells, tumor cells, and epithelial cells exhibited a unique signature. These results suggest that CDK1 mainly exerts biological effects in the TME by affecting the interaction between macrophages, T cells, and tumor cells.

In conclusion, CDK1 is a promising member of the CDK family in LUAD and plays an essential role in tumor cell proliferation and immune microenvironment regulation. It is extremely important to emphasize the differences in CDK1 expression in different patients to optimize treatment methods and accurately evaluate prognosis.

## Data availability statement

The datasets presented in this study can be found in online repositories. The names of the repository/repositories and accession number(s) can be found in the article/**Supplementary Material**.

## Author contributions

Conceptualization, DH and TQ. Methodology, QD, WL and JW. Software, QD. Validation, TQ. Formal analysis, QD. Writing - Original Draft, QD and WL. Writing - Review and Editing, DH and TQ. Visualization, TM. All authors have read and agreed to the published version of the manuscript. All authors contributed to manuscript revision, read, and approved the submitted version.
