# Supplementary figures and images for: Prognostic and immunological characteristics of CDK1 in lung adenocarcinoma: A systematic analysis
Source: Front Oncol. 2023 Mar 6;13:1128443. doi: 10.3389/fonc.2023.1128443 (PMC10025485; doi:10.3389/fonc.2023.1128443)

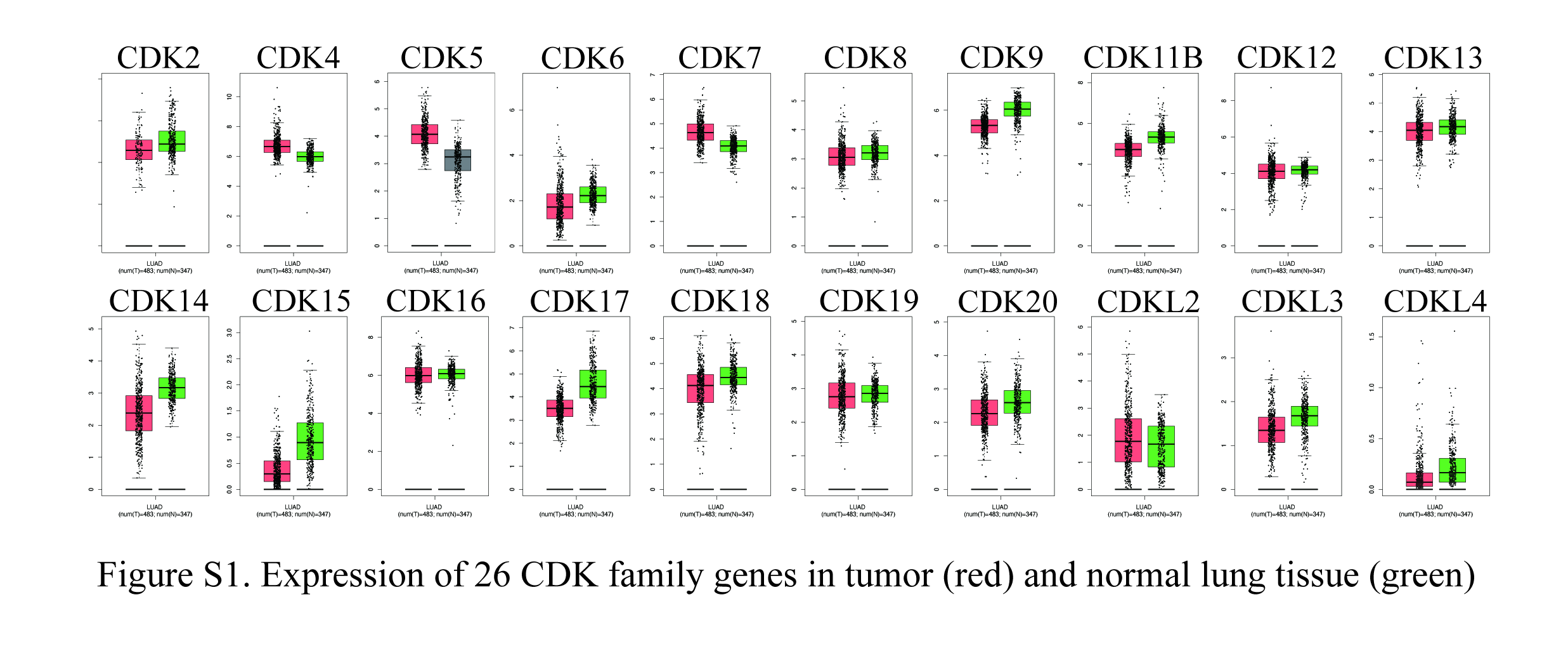

Supplement: Supplementary file 1 [file Image_1.tif]

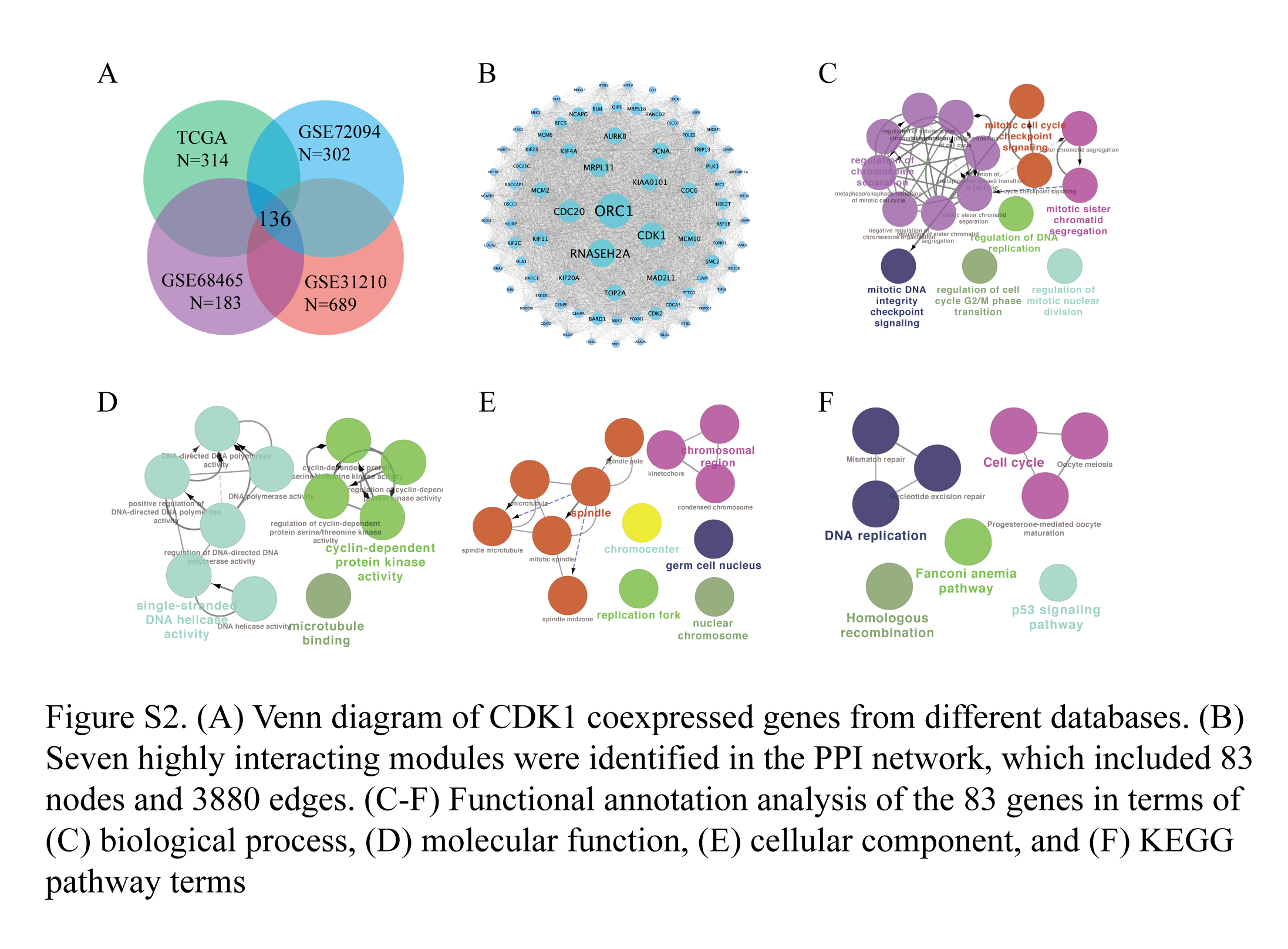

Supplement: Supplementary file 2 [file Image_2.tif]

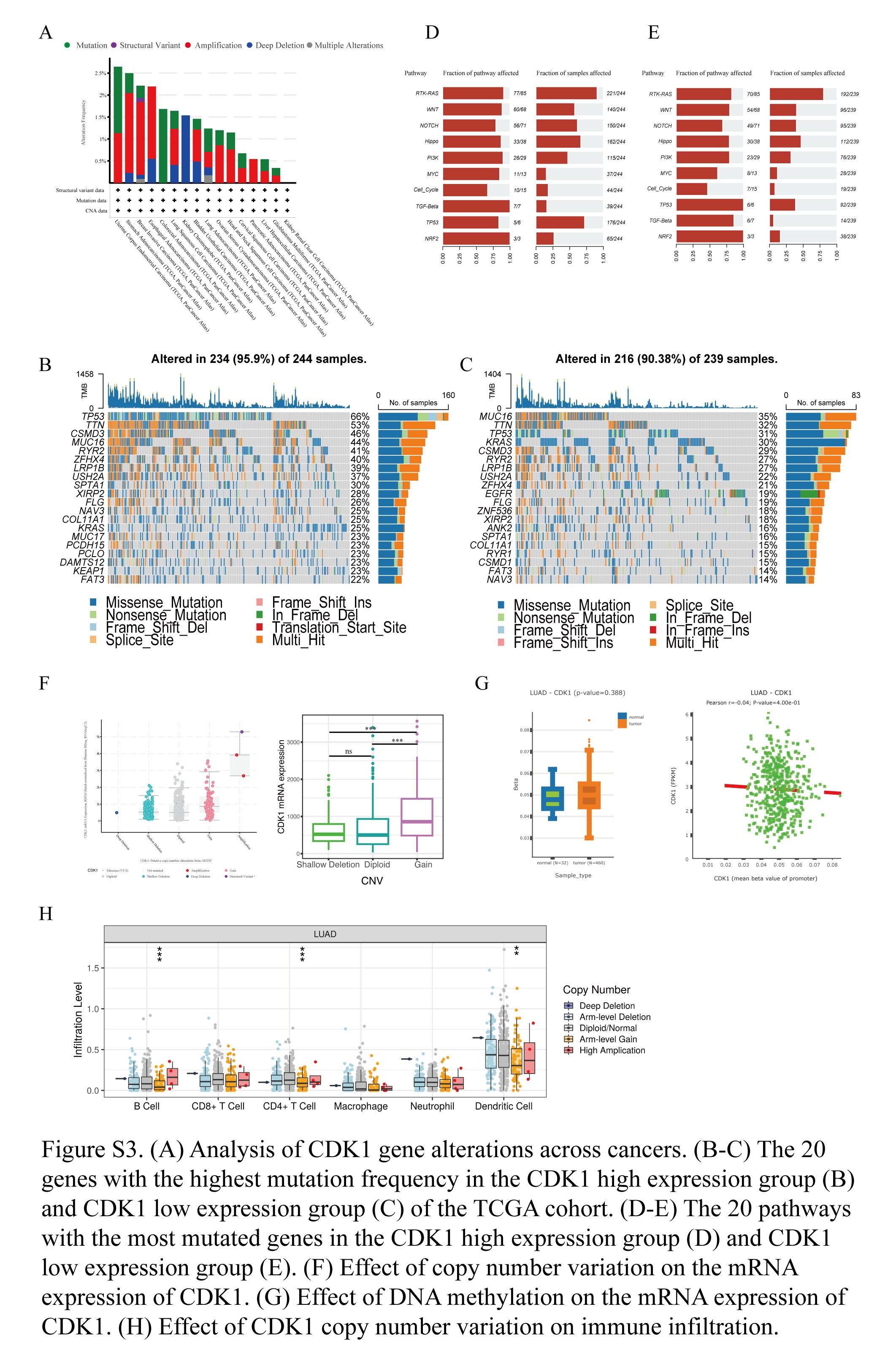

Supplement: Supplementary file 3 [file Image_3.tif]

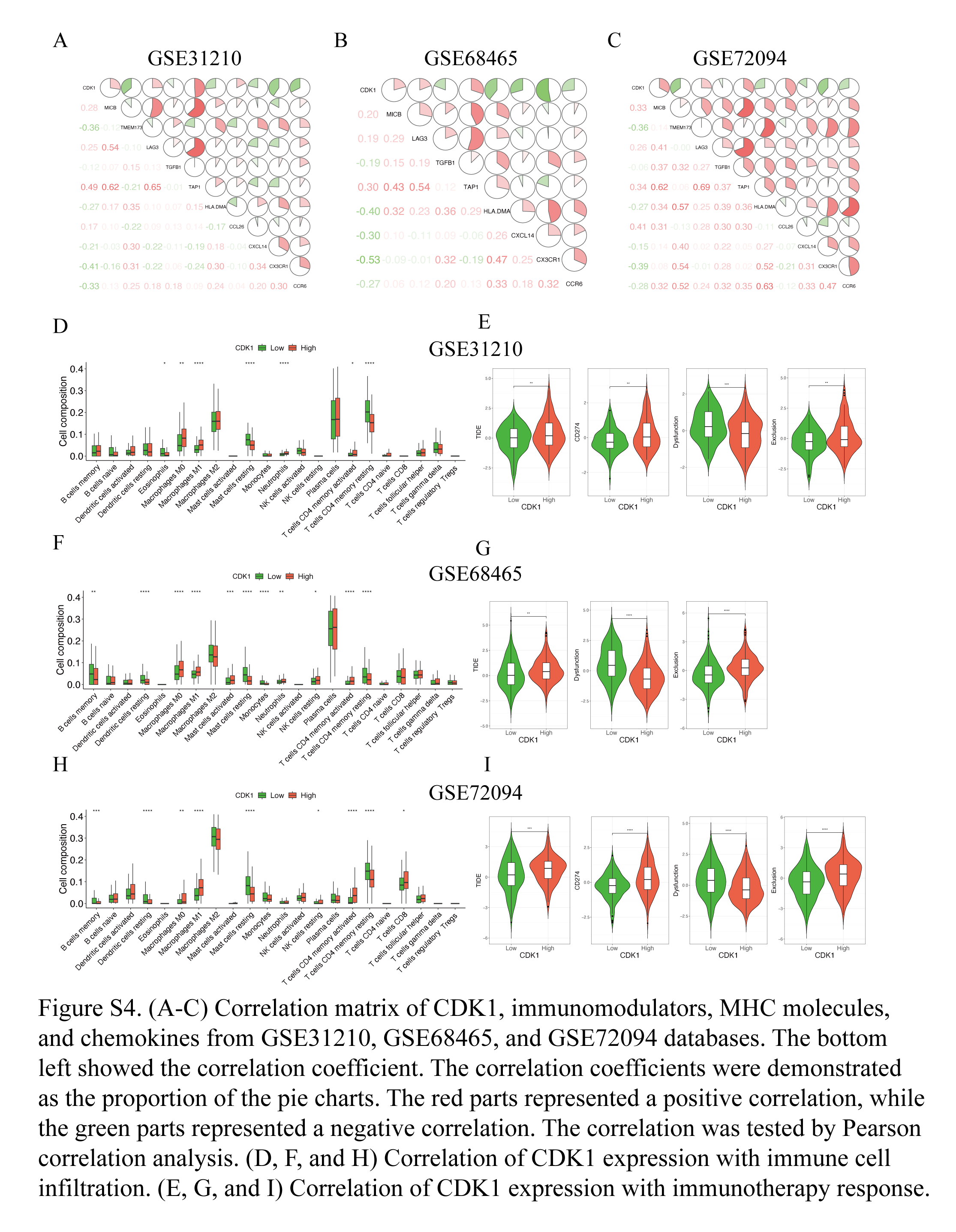

Supplement: Supplementary file 4 [file Image_4.tif]

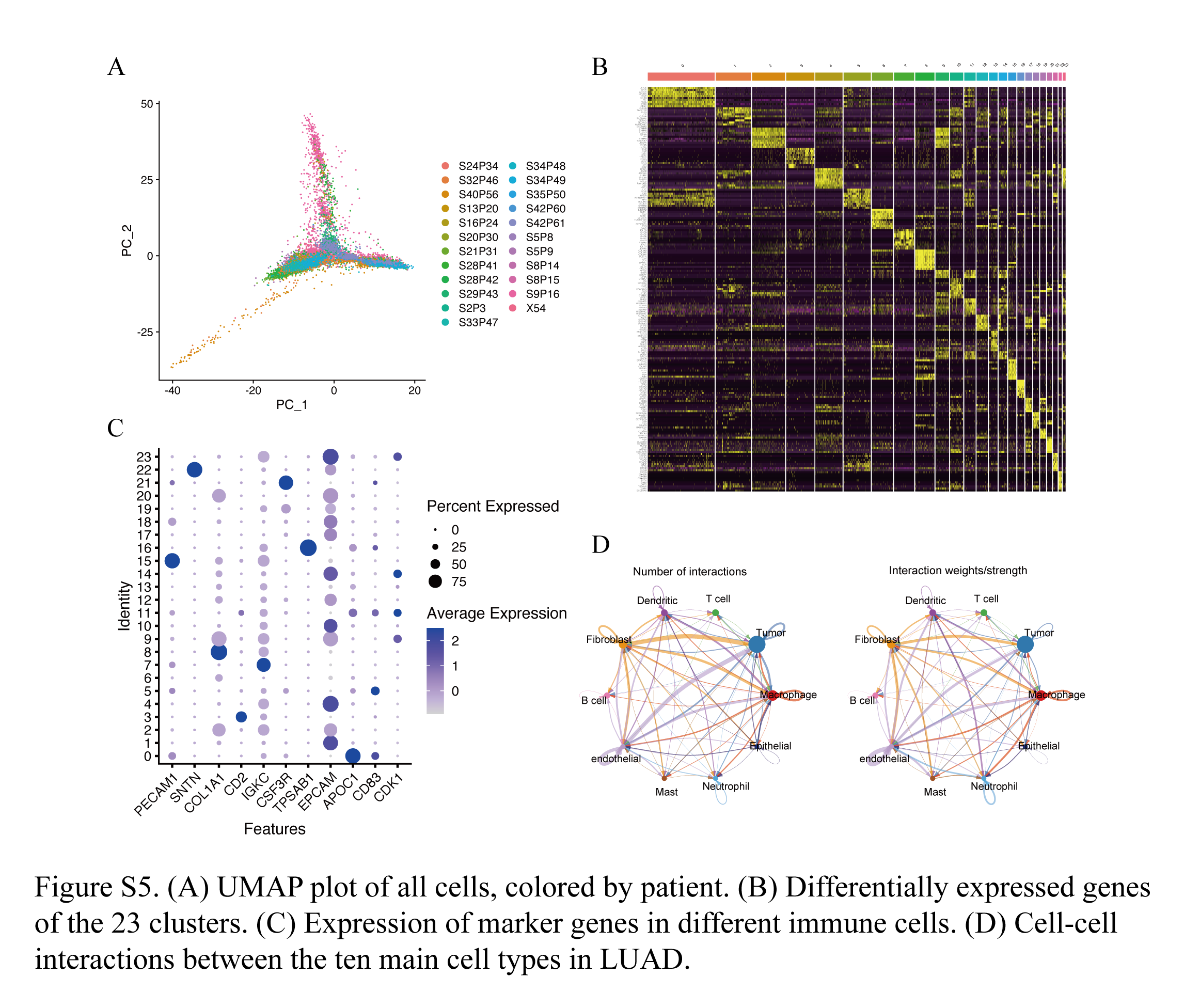

Supplement: Supplementary file 5 [file Image_5.tif]
